# Exploring Patient Pathways and Care Situations in Men With Erectile Dysfunction in Different PDE-5 Inhibitor Regulatory Settings

**DOI:** 10.3389/ijph.2025.1608529

**Published:** 2025-12-15

**Authors:** Uwe May, Quirin Werthner, Harald Weigmann, Cosima Bauer

**Affiliations:** 1 Faculty of Economics and Management, Fresenius University of Applied Sciences, Wiesbaden, Germany; 2 Independent Researcher, Darmstadt, Germany; 3 Opella, Frankfurt amMain, Germany; 4 May & Bauer GmbH & Co. KG, Bad Honnef, Germany

**Keywords:** erectile dysfunction, over the counter, PDE-5 inhibitors, pharmacy, prescription

## Abstract

**Objectives:**

The study examined the impact of varying prescription requirements for phosphodiesterase-5 inhibitors (PDE-5is) on the management of erectile dysfunction (ED) in men.

**Methods:**

A survey involving 10,000 men from Germany, Norway, Poland, and Switzerland was conducted using the International Index of Erectile Function Questionnaire to identify men with ED and interview them regarding their treatment and experiences. The use of PDE-5is by men without ED was also investigated.

**Results:**

The proportion of PDE-5i users with ED was higher in Norway and Poland (over-the-counter availability) and Switzerland (prescribed by pharmacist) than that in Germany (doctor’s prescription required). Across all countries, men seeking to buy PDE-5is from pharmacies were advised to consult a doctor. The use of black market channels for purchasing PDE-5is was minimal in regions allowing non-prescription availability. The non-medical use was uncommon in all regions.

**Conclusion:**

The need for a prescription for PDE-5is may hinder seeking treatment for ED and detecting underlying conditions. Therefore, PDE-5is should be approved as prescription-free medicines to improve the management of ED and to encourage men’s engagement with the healthcare system.

## Introduction

The World Health Organization has emphasized the importance of sexual health, highlighting its critical role in enhancing the physical and emotional wellbeing of individuals, couples, and families; nevertheless, men’s health has been given limited attention [1, 2]. Since 2009, male health conditions, such as erectile dysfunction (ED), have garnered attention, when a link between ED in young men and increased future cardiovascular (CV) risk was reported. ED is characterized as the inability to achieve and maintain an erection sufficient for satisfactory sexual intercourse, which can significantly impact an individual’s sexual performance and overall wellbeing [1, 3]. Data from the National Health and Social Life Survey have revealed that sexual dysfunction is linked to poor physical and emotional health and decreased quality of life (QoL) [4, 5]. Moreover, ED frequently occurs because of CV diseases, endocrine disorders, or depression [6], and in individuals those with chronic diseases as well as in those who are apparently healthy [7].

Phosphodiesterase-5 inhibitors (PDE-5is) have been designated as a first-line therapy for ED, endorsed by both the American Urological Association and the European Association of Urology [8]. Several PDE-5is, such as sildenafil (Viagra since 1998), tadalafil (Cialis since 2003), vardenafil (Levitra since 2003), and avanafil (Stendra since 2012), are approved for the treatment of ED [8].

Despite the high prevalence of ED and other health issues, many men avoid treatment due to various inhibitions and barriers, including preferences and knowledge among physicians, patients, and partners [9]. Many men find it challenging to consult a doctor and to admit to having ED. Over 70% of ED cases remain undiagnosed because of the reluctance of men to discuss it, despite its significance as a global health issue [10]. This reduces the opportunities for receiving preventive care and proper treatment for ED and other underlying conditions [5, 11].

To the availability of PDE-5is is a critical factor in determining their use in the treatment of ED. Sildenafil and/or tadalafil can be acquired from pharmacies without a prescription in several countries, such as Poland, Norway, New Zealand, Ireland, and the United Kingdom. However, in most of the other countries, PDE-5is are categorized as prescription medicines [12]. Patients also acquire PDE-5is via illegal methods due to restricted access [13, 14], bypassing the authorized healthcare system, thereby avoiding screenings for comorbidities.

Thus, treatment behaviors for ED should be investigated to seek further insights into ED treatment and also unravel information regarding other potentially serious but undetected ED-related health issues, such as diabetes, hypertension, coronary artery disease, and depression, at an early stage.

This study collected comprehensive data on the prevalence of ED and the behavior of men in dealing with ED. We investigated whether access to PDE-5is, based on varying prescription requirements, impacted the care and treatment of men affected by ED. We compared countries with different prescription status, such as prescription (Rx) by a doctor (Germany, DE), non-prescription (over the counter [OTC]) (Poland, PL; Norway, NO), and prescription by a pharmacist (Switzerland, CH), to assess the different regulatory approaches regarding benefits and risks for patients with ED. This analysis elucidated both treatment rates and the possibility of early diagnosis of underlying conditions, such as CV diseases.

## Methods

### Study Design

A representative survey was conducted between April 14, 2023 and May 5, 2023, including participants from four countries: DE, NO, PL, and CH. A total of 10,000 men from these four countries underwent screening.

### Participant Selection

A slightly higher number of participants than needed were invited from the online panels to answer the questionnaire based on quotas, which followed the national representative structure according to Eurostat (2021 data). Quota were based on the distribution of gender and region among males aged 18–75 years in each country. Consent to participate in the survey (General Data Protection Regulation consent to data collection) was obtained from the participants before administering the questionnaire. The participants agreed to record personal data related to their health and sexual life and granted permission to share information regarding adverse events (AEs) and product quality complaints with the sponsor. After screening, the eligible respondents were given the main survey questionnaire. The main questionnaire included the following parameters: identification of men experiencing ED, therapy seeking and consideration of treatments for ED symptoms, consultations with healthcare professional (HCP) (doctor/pharmacist) for ED-related topics, examination or therapies received to treat ED symptoms, reasons for not consulting a doctor, the use of PDE-5is, and the channels through which men accessed these treatments. Recruitment was stopped upon reaching the required number of participants across various regions, age groups, and other demographic groups.

### Sampling and Data Collection

**FIGURE 1:**
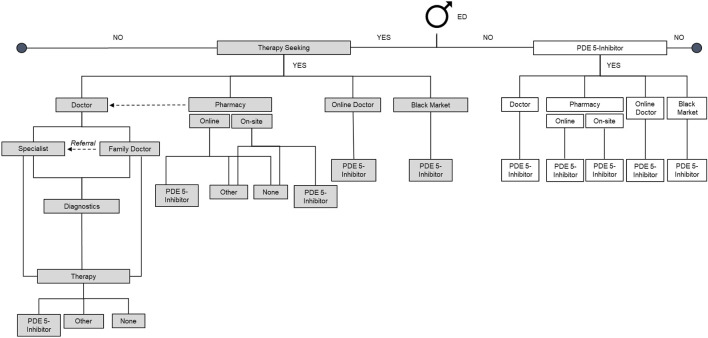
Decision tree (Germany 2025). ED, erectile dysfunction; PDE-5i, phosphodiesterase-5 inhibitor.

A sampling software identified respondents from the panel that matched the target criteria to represent the male population. The panelists were sent a general invitation that was directed to a router to enter the survey. They could also access the surveys through the panelist website or the application dashboard. Patient behavior in the ED category and the quantification of decision tree paths were also analyzed (Figure 1).

The participants were interviewed for 12 min, on average. Additionally, any AEs related to the products were recorded.

### Scoring

The presence of ED was evaluated using the International Index of Erectile Function (IIEF-5) questionnaire, entailing five questions, each rated on a scale of 0–5 (first question 1–5 points), for a total score ranging from 0 to 25 points. ED was identified if the IIEF-5 score was ≤21. The degree of ED, as defined by the IIEF-5 score, was categorized as follows: mild, 17–21; mild-to-moderate, 12–16; moderate, 8–11; and severe: 1–7 (Supplementary Tables S1, S2) [15, 16]. Furthermore, all men were asked to complete a self-assessment through a closed question to determine whether they experienced ED (self-reporter).

### Outcome Measures

Patient groups were defined based on the data collected according to the requirements of a decision analytic model. The prevalence of ED was determined as the percentage of participants who either self-reported experiencing ED or were classified as having ED according to the IIEF-5 scoring system.

### Statistical Analysis

Quota sampling using Eurostat (2021 data) ensured a nationally representative distribution by age and region in DE, NO, and CH. In PL, the data were weighted in terms of age distribution to align with the Eurostat data, as individuals aged 61–75 years were underrepresented. This survey weight (rim weighting efficiency, 99.2%) was used in all computations for PL. The data were analyzed using descriptive statistics, logistic regression, and Chi-square tests to determine group differences for dichotomous variables. Ordinal logistic regressions were performed for age groups. Furthermore, multinomial regression was used to analyze the relationship between men with ED and the availability of OTC medication. All regression analyses were adjusted for age and country. Missing data in the regression analyses were deleted listwise. P-values and odds ratios (ORs) with confidence intervals (CIs) were reported for each regression, with p < 0.05 considered significant. All statistical analyses were conducted using R version, whereas the weighting of data was conducted using SPSS.

## Results

### Prevalence of ED

**FIGURE 2:**
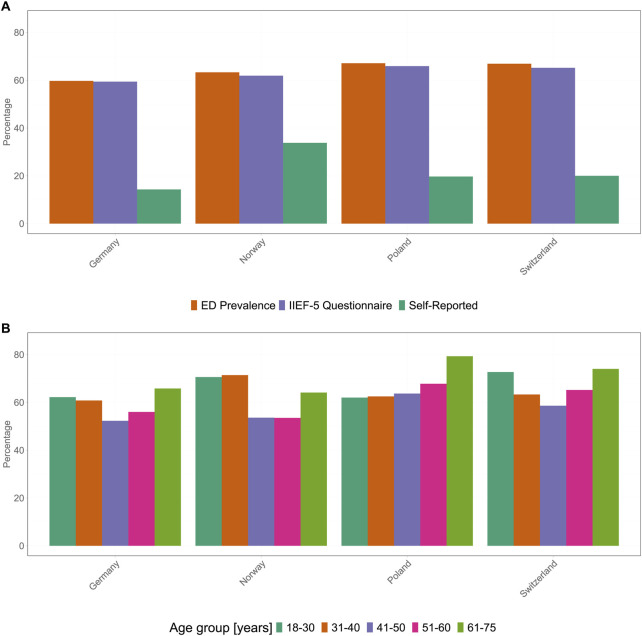
Incidence of erectile dysfunction in four different countries. **(A)** Erectile dysfunction prevalence (orange), as assessed by the International Index of Erectile Function-5 scoring system (purple) and erectile dysfunction self-reported by participants (dark green). **(B)** Distribution of erectile dysfunction prevalence in the four countries by age group: dark green, 18–30 years; orange, 31–40 years; purple, 41–50 years; pink, 51–60 years; and light green, 61–75 years (Germany 2025). ED, erectile dysfunction; IIEF-5, International Index of Erectile Function.

Across all four countries, 10,000 respondents completed the questionnaire, with 2,500 respondents per country. The average age of the participants was similar in all four countries, with mean ages being 46.3, 45.1, 45.0, and 44.8 years in DE, NO, PL, and CH, respectively. In the present study, 1,493 (DE), 1,582 (NO), 1,665 (PL), and 1,672 (CH) participants with ED were included. The prevalence of ED varied between the four countries depending on the assessment method. Overall, ahigh prevalence of ED was observed in all four countries (DE, 59.7%; NO, 63.3%; PL, 67.1%; and CH, 66.9%). Similarly, the proportions of the participants being diagnosed with ED based on the IIEF-5 scoring were comparable in all four countries (DE, 59.4%; NO, 61.9%; PL, 65.9%; and CH, 65.2%). In contrast, self-reported ED cases were more common in NO (33.8%, p < 0.001), PL (19.7%, p < 0.001), and CH (20.0%, p < 0.001) than in DE (14.3%) (Figure 2A).

### Prevalence of ED in Different Age Groups

In all four countries, the prevalence of ED was high in all age groups, with younger and older individuals exhibiting a higher prevalence than that of the middle-aged individuals (Figure 2B). In DE, the prevalence of ED was the highest in participants aged 61–75 years (65.8%), followed by participants aged 18–30 years (62.2%) and 31–40 years (60.8%). The prevalence of ED was lower in participants aged 51–60 years and 41–50 years, at 56% and 52.3%, respectively. Although the overall prevalence was slightly higher in CH, its trend was similar to that observed in DE, with the highest prevalence in participants aged 61–75 years (74.0%), followed by participants aged 18–30 years (72.7%) and 51–60 years (65.2%); the prevalence of ED was the lowest in participants aged 31–40 years (63.3%) and 41–50 years (58.6%). In PL, the prevalence of ED increased with age, and the highest prevalence was observed in participants aged 61–75 years (79.3%), followed by participants aged 51–60 years (67.8%). The prevalence of ED was 63.7%, 62.5%, and 62.0% in individuals aged 41 to 50, 31 to 40, and 18–30 years, respectively. Finally, the prevalence of ED in NO was similar to that in DE and CH, with the lowest being in the middle-aged groups, i.e., 53.5% for participants aged 51–60 years and 53.6% for those aged 41–50 years. In contrast to the other age groups, the highest ED prevalences in NO were observed in the younger age groups: 71.4% in participants aged 31–40 years and 70.6% in the youngest age group (18–30 years). In the oldest age group in NO, 64.1% of participants (61–75 years) were classified as experiencing ED.

### Severity of ED

**FIGURE 3:**
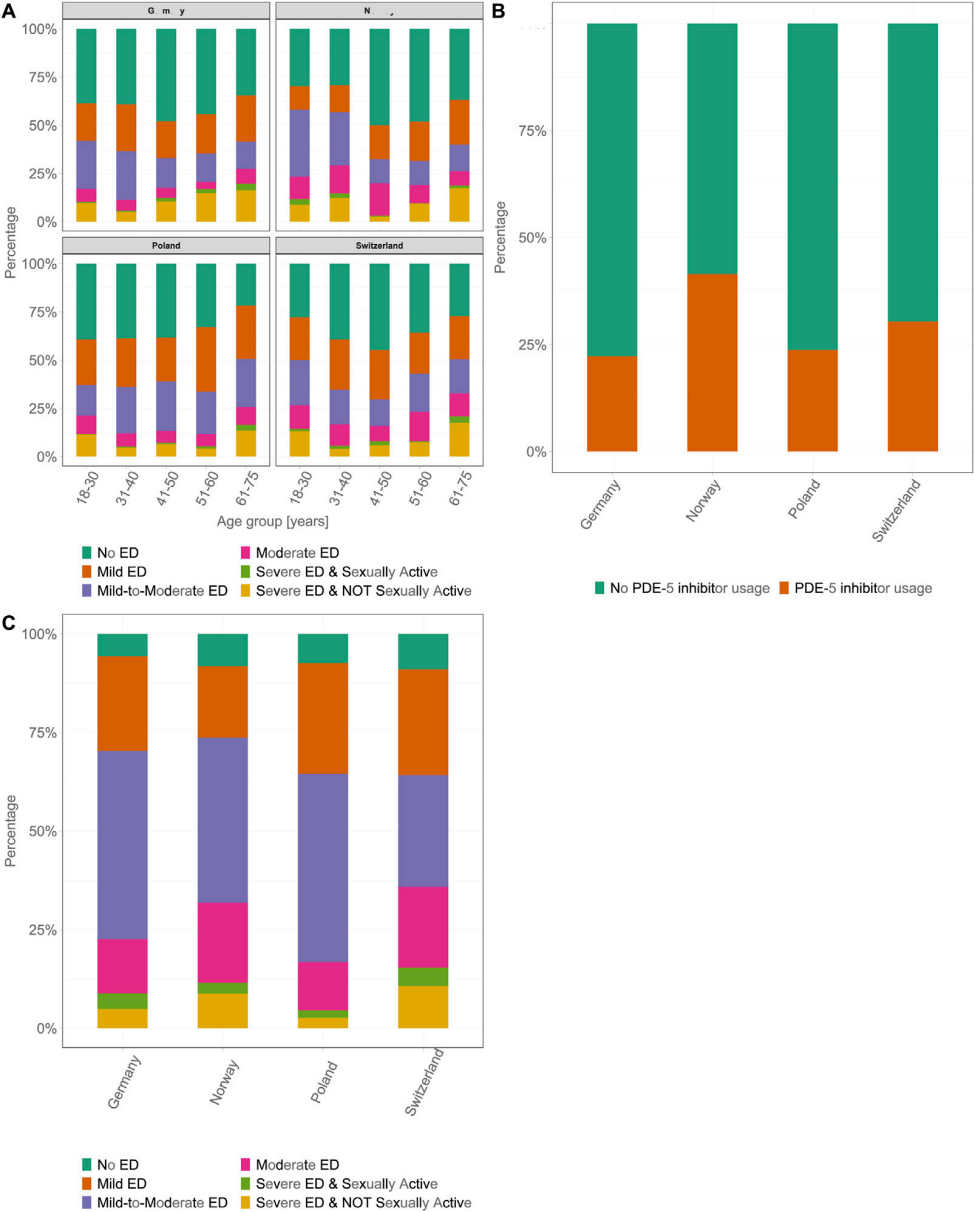
Country-wise stages of erectile dysfunction severity and phosphodiesterase-5 inhibitors usage. **(A)** Prevalence and severity of erectile dysfunction across six age groups (18–30, 31–40, 41–50, 51–60, 61–75 years) for each country. The severity categories are color-coded as follows: no erectile dysfunction, dark green; mild erectile dysfunction, orange; mild-to-moderate erectile dysfunction, light green; moderate erectile dysfunction, purple; severe erectile dysfunction and sexually active, pink; and severe erectile dysfunction and not sexually active, yellow. **(B)** Percentage of participants with erectile dysfunction who used phosphodiesterase-5 inhibitors compared with those who did not use phosphodiesterase-5 inhibitors. Data are represented as stacked bars for each country, with green and orange indicating no phosphodiesterase-5 inhibitors usage and phosphodiesterase-5 inhibitors usage, respectively. **(C)** Country-wise distribution of erectile dysfunction severity for participants on phosphodiesterase-5 inhibitors therapy. The bars are divided into different categories of erectile dysfunction severity; color-coding same as that used in panel **(A)** (Germany 2025). ED, erectile dysfunction; PDE-5i, phosphodiesterase-5 inhibitor.

The severity of ED, as assessed by the IIEF-5 scoring system, showed a similar pattern in all four countries. The majority of patients had mild or mild-to-moderate ED, regardless of age. The least common grade of severity was “severe ED,” which occurred in ≤4% of participants in all age groups across all four countries. Overall, the pattern of ED severity was consistent throughout the four countries and was similar to that of the prevalence of ED across all age groups. Higher severity levels were more common in the youngest age group (18–30 years) than in participants aged 31 to 40 and 41–50 years. Consequently, the frequency of higher severity levels increased again in the older age groups (Figure 3A). Despite similar ED prevalence rates across the four countries, there were significant differences in the proportion of participants receiving therapy. In DE, 22.2% of participants with ED used a PDE-5i. In contrast, higher proportions of participants in NO and CH were likely to receive PDE-5i therapy, with 30.4% (OR 1.54, 95% CI [1.31, 1.80], p < 0.001) in CH and 41.5% (OR 2.34, 95% CI [2.01, 2.73], p < 0.001) in NO. In PL, 24.1% of participants were using PDE-5is, which was higher than those in DE, but this difference was not statistically significant (Figure 3B). The majority of patients on PDE-5i therapy were classified as having mild-to-moderate ED. In NO and CH, the rates of participants classified as having “severe ED and not sexually active” (NO: 8.8%, CH: 10.7%) were higher than those in PL (2.3%) and DE (4.9%). A similar pattern was observed in the “severe ED and sexually active” category (NO: 2.7%, CH: 4.7%, PL: 1.9%, DE: 4.0%) (Figure 3C).

In DE, 39.3% of participants with ED had consulted a doctor about erection problems or unsatisfactory sexual performance; however, higher rates of doctor consultations were recorded in NO (59.6%, OR 2.25, 95% CI [1.94, 2.60], p < 0.001) and CH (44.3%, OR 1.21, 95% CI [1.05, 1.40], p = 0.008). In PL, only one in four participants consulted a doctor about their condition (25.0%, OR 0.51, 95% CI [0.44, 0.60], p < 0.001).

### Management of ED

**FIGURE 4:**
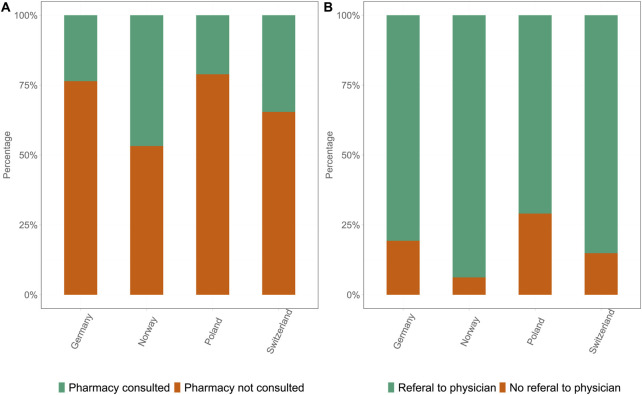
Proportion of pharmacy consultations and physician referrals in all four countries: Germany, Norway, Poland, and Switzerland. **(A)** Proportion of participants who consulted a pharmacist (green) versus those who did not consult a pharmacist (orange). **(B)** Proportion of participants who were referred by the pharmacist to a physician (green) versus those who were not referred to a physician (orange) (Germany 2025).

The data revealed marked differences in the utilization of HCPs for the management of ED in all four countries. The proportion of participants who consulted a pharmacist for ED management was significantly different between the four countries (Figure 4A). The percentage pf participants who consulted a pharmacist were 23.6% in DE and 21.5% in PL. When additionally correcting for self-reported suffering and being classified as having ED, NO and CH showed higher consultation rates, with 46.8% (OR 2.00, 95% CI [1.72, 2.34], p < 0.001) and 34.6% (OR 1.57, 95% CI [1.33, 1.85], p < 0.001) of participants, respectively, consulting a pharmacist. The majority of pharmacists referred the participants experiencing ED to physicians for further checkups in accordance with common practice.

### Physician Referral

The referral rates also varied between the countries. Similar patterns were observed for consultations with a pharmacist (Figure 4B). In DE, 80.7% of participants who consulted a pharmacist were also referred to a physician, whereas in PL, the referral rate was only 70.6% (OR 0.61, 95% CI [0.43, 0.85], p = 0.0039). In CH and NO, 85.1% (OR 1.44, 95% CI [1.02, 2.05], p = 0.04) and 93.8% (OR 3.35, 95% CI [2.30, 4.87], p < 0.001) of participants, respectively, received a referral from their pharmacist.

The use of PDE-5is without an indication, i.e., in individuals who neither self-reported ED nor met the criteria for ED as per the IIEF-5 scoring system, was rare in all four countries. In DE, only 18 participants who did not experience ED used PDE-5is. A similar trend was observed in NO, PL, and CH, with 39, 24, and 25 participants, respectively, who used PDE-5is without a clinical indication.

### Various Channels for Acquiring PDE-5is

**FIGURE 5:**
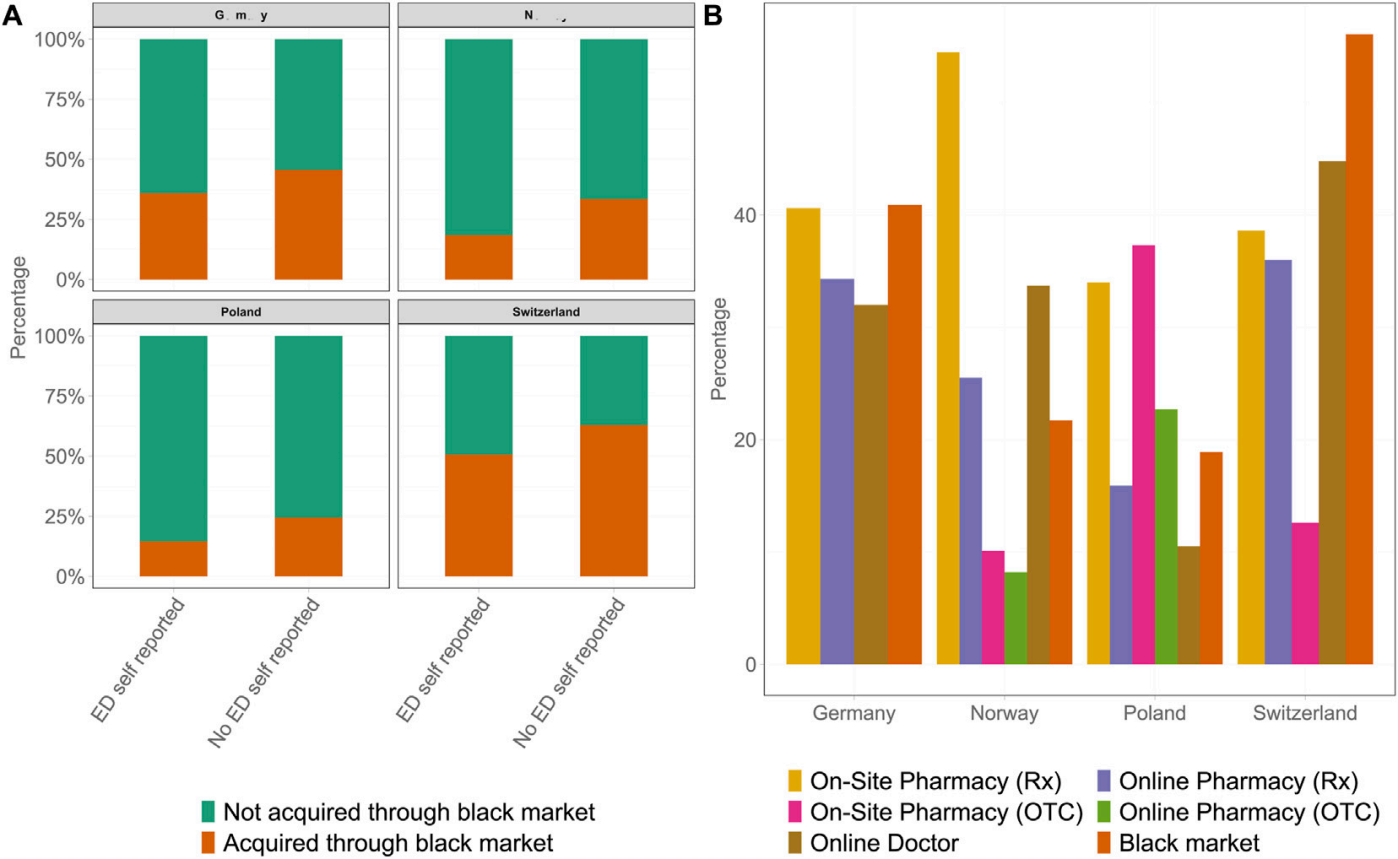
Comparative analysis of methods used for acquiring phosphodiesterase-5 inhibitors for erectile dysfunction treatment across all four countries. **(A)** Rates of drug acquisition through the black market for self-reported participants with erectile dysfunction (left bar) versus those diagnosed with erectile dysfunction via the International Index of Erectile Function scoring system. Participants who did not acquire phosphodiesterase-5 inhibitors via the black market are shown in green, whereas black market acquisition is depicted in orange. **(B)** The sources of phosphodiesterase-5 inhibitors: on-site pharmacies with Rx in yellow, on-site pharmacies over the counter in pink, online pharmacies with Rx in purple, online pharmacies over the counter in green, online doctors in brown, and the black market in orange. Each bar represents the percentage of participants using each method in the respective country (Germany 2025). ED, erectile dysfunction; IIEF-5, International Index of Erectile Function; OTC, over the counter; PDE-5i, phosphodiesterase-5 inhibitor.

Logistic regression, additionally correcting for ED severity and doctor consultation, indicated that self-reporting was associated with a lower likelihood of black market use (Figure 5A). In a weighted multinomial regression with legally sourced PDE-5i as the reference category, country of residence, age, doctor consultation, and ED severity were significantly associated with treatment patterns. Respondents from Norway (21.7%, OR = 0.43, 95% CI: 0.29–0.63, p < 0.001) and Poland (18.9%, OR = 0.21, 95% CI: 0.13–0.35, p < 0.001) had substantially lower odds of using the black market compared with those from Germany (40.9%), whereas respondents from Switzerland were more likely to report the black market use (56.1%, OR = 1.65, 95% CI: 1.10–2.46, p = 0.01). Similar associations were observed for participants who self-reported having ED, with men from Norway (OR = 0.59, 95% CI: 0.43–0.80, p < 0.001) and Poland (OR = 0.39, 95% CI: 0.28–0.56, p < 0.001) less likely to remain untreated relative to legal users. Age was also related to the use of the black market. Men aged 31–40 years (OR = 1.55, 95% CI: 1.04–2.32, p = 0.03) and 41–50 years (OR = 1.83, 95% CI: 1.20–2.79, p = 0.005) were more likely to report the black market use than men aged 18–30 years, whereas older age groups (51–75 years) did not differ significantly. Doctor consultation was a strong predictor of legal use. Men who had consulted a doctor had markedly lower odds of both black market use (OR = 0.43, 95% CI: 0.31–0.60, p < 0.001) and remaining untreated (OR = 0.14, 95% CI: 0.11–0.18, p < 0.001) relative to legal use. Finally, ED severity showed no significant associations with the use of the black market. In contrast, participants classified as having mild ED (OR = 0.44, 95% CI: 0.26–0.74, p = 0.002), mild-to-moderate ED (OR = 0.36, 95% CI: 0.22–0.60, p < 0.001), and moderate ED (OR = 0.43, 95% CI: 0.31–0.60, p = 0.005) showed higher odds of remaining untreated even though they self-reported as suffering from ED. Furthermore, the study revealed other differences in the specific sources that were used to acquire ED medication (Figure 5B). Although the easiest access to PDE-5is was observed in NO, the highest rate of on-site pharmacy utilization with a prescription was also observed in NO at 54.5%. Furthermore, other physician-based ways of access were often used with online doctors (33.7%) and online pharmacies (25.5%), which were the second and third most frequent ways of receiving ED medication, respectively. Approximately 21.7% of participants acquired medication from black market channels, whereas OTC options, both on-site and online pharmacies, were only used by 10.1% and 8.2% of participants, respectively.

### OTC Availability of PDE-5is

In DE, no OTC PDE-5is are available, and this class of medication remains available by prescription only; thus, black market channels were the predominant source of acquiring ED medication (40.9%), followed by on-site pharmacies with prescription (40.6%), online pharmacies (34.3%), and online doctors (32.0%). In PL, the distribution of sources was more varied. On-site pharmacies were the main channel for acquiring PDE-5i, with 34.0% and 37.3% of participants receiving medication with a prescription and OTC, respectively. Furthermore, 16.2%, 22.8%, and 19.0% of participants acquired medication via online pharmacies with prescription, online pharmacies without a prescription, and black market channels, respectively; only 10.8% of participants who received PDE-5i-based therapy consulted online doctors. In contrast, the highest rate of black market use and the highest proportion of participants who consulted an online doctor (44.8%) were observed in CH, whereas the use of prescription channels, such as on-site pharmacies with a prescription (38.6%) as well as online pharmacies (36.0%), was similar to that observed in DE. Only 12.6% of participants utilized the OTC on-site pharmacies.

## Discussion

**FIGURE 6:**
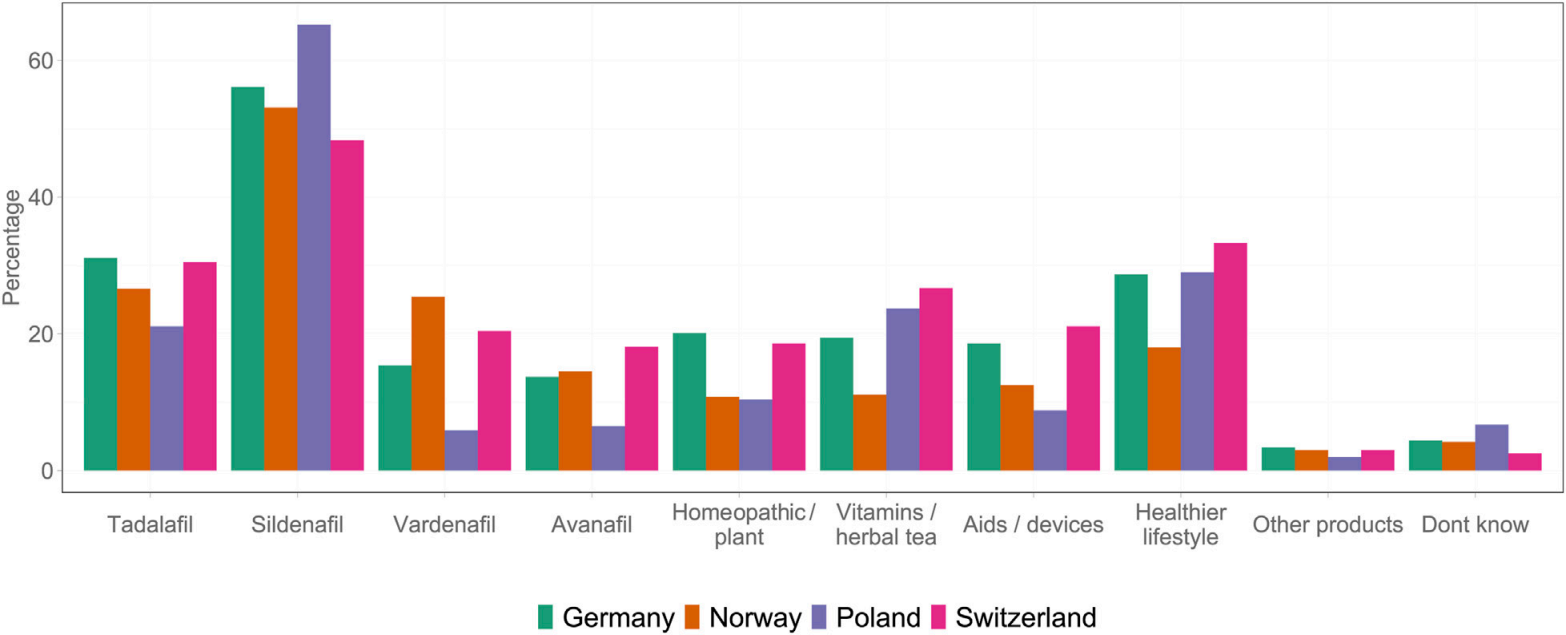
Summary of all the products used by the participants across four countries (Germany 2025).

In this study, the prevalence of ED, assessed through an internationally established diagnostic questionnaire (the IIEF-5 scoring system), was similar across all four countries and all age groups. However, patient journey, i.e., the path to therapy and successful treatment, varied significantly between these countries; this could be because of the difference in the prescription status of PDE-5is in these countries. The prevalence of ED observed in this study was similar to the rates (61.1%) reported in another study conducted in PL [17]. However, lower rates (12%–48%) have also been observed among individuals in Finland [18]. The variations in the prevalence of ED in different studies could be due to differences in methodology and subjective perceptions. The proportion of self-reporters was lower than the proportion of participants diagnosed using the IIEF diagnostic questionnaire. Some of the previous studies have also reported using IIEF-5 for detecting the presence and severity of ED, as it is an internationally used and validated questionnaire owing to its accuracy. The studies have reported a significant correlation (Spearman r = 0.80) between the questions in the questionnaire and the results of independent urologic examinations [19] and the clinical evaluation and the diagnosis of ED [15]. Furthermore, the proportion of self-reporters was significantly higher in countries where doctor prescriptions are not mandatory for PDE-5is. This suggests that the availability of OTC medicines leads to a more open social approach to ED. The availability of PDE-5is has the potential to significantly enhance the wellbeing of men, influencing various aspects, such as the number of individuals actively seeking treatment, their interactions with healthcare providers, and their engagement with pharmacies. Physician referrals by pharmacists for ED-related consultation were more prevalent in countries where PDE-5is were available OTC. These findings suggest that the OTC availability of ED medications may support appropriate treatment uptake among men with a diagnosis. In Norway and Poland, where OTC access exists, men were less likely to turn to the black market or remain untreated and more likely to rely on legal sources. In contrast, Switzerland, with high prices and pharmacy prescription requirement combined with a high mandatory consultation fee at the pharmacy, showed higher black market use. The conclusion that the treatment rate is higher under OTC conditions than under prescription requirements is based not only on the comparison between countries but also on further findings. In our survey itself, we also asked untreated men in our reference country with prescription status (Germany) about the obstacles they believed were hindering access to therapy. Practical barriers (e.g., time required) and also psychological barriers that prevent them from visiting a doctor were frequently mentioned. Furthermore, those affected by ED were asked whether they would be more likely to use PDE-5i preparations if they were available OTC and whether purchases on the black market would decline. A significant proportion of men responded affirmatively to both questions. Beyond our survey, the market data from countries where a switch has taken place (e.g., Norway and Ireland) also show that OTC availability has led to an increased use of the preparations in the respective country [20]. These findings are also consistent with general research results in other areas of healthcare, which show that low-threshold access to care in pharmacies promotes the use of healthcare services. This has been empirically proven particularly well in the case of vaccination services in pharmacies. This study also shows that often time and psychological barriers that prevent people from visiting a doctor’s office [21]. Consultations with doctors promoted strong legal use of PDE-5is (irrespective of the country), emphasizing the importance of medical contact. The results support the view that lowering access barriers through OTC availability might discourage unregulated use and improve treatment coverage in populations with a demonstrated need (Figure 6).

The data suggest that ED is associated with CV diseases; thus, patients should be advised to consult a doctor to mitigate the risk of CV diseases [13, 22]. Moreover, pharmacists should initially inquire about CV health, concurrent medication usage, and any coexisting health conditions. They should recommend men purchasing PDE-5is to follow up with their doctor within 6 months, or sooner, to ensure the investigation of any underlying health conditions [23]. This study demonstrated that in all four countries, the majority of men considering the purchase of a PDE-5i from a pharmacy were advised to consult a doctor (DE: 81%; NO: 94%; PL: 71%; CH: 85%). This trend existed in both cases, i.e., where a doctor’s prescription was mandatory, such as, in DE (81%), and where prescriptions by medical doctors were not required, such as, in NO (94%), CH (85%), and PL (71%). In many European nations, pharmacies are highly trusted by the public, and their recommendations are frequently followed [24, 25], suggesting that although PDE-5is are accessible without a prescription in these countries, individuals with ED follow medical advice to see a doctor. This supports the fact that in NO, where PDE-5is are OTC medicines with a low-threshold for access, the proportion of PDE-5i prescriptions, both by a doctor and at an on-site pharmacy (80%), was higher than that in DE and other reference countries. A previous study analyzed possible changes to the characteristics of the Individual Case Safety Reports, such as seriousness or outcomes of reported AEs using tadalafil in countries where it was switched from prescription to OTC. The results demonstrated that the usage patterns of tadalafil did not cause incidents of reported AEs or any other safety signal that is possibly associated with switching to OTC [26]. Another publication analyzing the benefits and risks of tadalafil as a non-prescription drug in Germany reported the consensus among clinical experts on the minimal incremental risks, if any, due to the switch to OTC. There was also a consensus that the incremental risks, if any, would be of little clinical impact, sufficiently manageable, and significantly lower than the potential benefits [22].

The challenges faced in accessing treatment for ED have led to a significant number of individuals resorting to the unauthorized use of PDE-5is, bypassing the conventional medical channels [11]. In 2010, a study reported that approximately 6 million men in Europe might be obtaining PDE-5is through channels outside the healthcare system [27]. According to our data, which corroborate with the literature, PDE-5is are being sourced from black market channels, particularly in those regions where the drug is not available OTC. Thus, it is imperative to approve PDE-5is as an OTC medicine to reduce the need for illegal sources and ensure safer, more accessible treatment options for patients. From the perspective of public health, it is important to recognize that the significance of illegal black market channels as a source for obtaining PDE-5is is much lower in countries where a prescription is not mandatory. In DE (Rx setting), more than every third participant (40.9%) reported illegal purchase of PDE-5is. In PL and NO (OTC setting), the proportion of black market use was significantly lower. However, a unique trend was observed in CH, where despite no prescription being required to obtain a PDE-5i, the use of black market channels was notably high. This can be attributed to the high healthcare costs in CH, where obtaining PDE-5is from a pharmacist involves a consultation fee, in addition to the already high price of OTC PDE-5is. As a result, many men in CH may be driven to seek more affordable alternatives through black market channels.

Instances of PDE-5i use without pre-existing medical indication were few across all four countries. One of the primary reasons for requiring prescriptions for PDE-5is is to control their use by individuals without ED. However, based on the data acquired through the survey, limited evidence supports this claim. In DE (Rx), only 18 out of 1,007 men who did not have ED used these drugs without a prescription, and a doctor prescribed the medication to the remaining men. Even in countries where PDE-5is are available without a prescription, individuals using them without ED indication were rarely observed (NO: 39/918; PL: 24/822; CH: 25/828). Moreover, in a previous study, the risks of using PDE-5is without ED indication were reported to be relatively low [19].

### Limitations

The study had a few limitations because the data from only a limited number of countries were analyzed, along with diverse cultural differences and variations in healthcare systems. This may have resulted in distinct treatment behaviors and varying degrees of QoL. It is often challenging to confirm the accuracy of the responses provided by the participants in questionnaire-based studies. The study reported that there could be a possible bias occurring from men concealing the fact they suffer from ED, and on the other hand, it is highly unlikely that healthy men would report suffering from ED. In the present study, the respondents are likely to describe their sexual performance as better, instead of worse, compared with the actual situation. This suggests that the prevalence and severity data collected in this study might have underestimated the actual disease burden of ED. Due to these conservative data on the prevalence of ED, there could be even more untreated men. Thus, strategies should be implemented to address this uncertainty, which will improve the reliability of the responses, contribute to more accurate representation of data, and reduce potential inaccuracies in data. The digital platform might also play a role in lowering the officially recorded statistics. Moreover, factors such as digital accessibility, internet literacy, and the nature of online interactions could influence the level of participation and response rates, thus influencing the overall data. Therefore, the above discussion emphasizes the need and urgency for a switch from prescription-only to OTC status of PDE-5is.

### Conclusion

ED is a highly prevalent condition in men, posing a substantial disease burden. In addition to the direct effects of ED on male sexual health, it is also linked to serious underlying conditions, such as coronary heart disease or diabetes. Despite its high incidence, ED is often considered a sensitive issue, potentially leading to it being frequently under-reported. This contributes to hesitation in seeking treatment, resulting in many patients remaining untreated. Against this background, we analyzed the effects of different legal frameworks regarding prescription requirements on patient pathways and the use of PDE-5is. The data obtained by comparing the practices followed in four different countries suggest that simplified access without a doctor’s prescription increased the proportion of patients using PDE-5is. Moreover, the proportion of users illegally sourcing PDE-5is through the black market was significantly lower in participants with OTC access (NO, PL) compared with DE, where these medicines are still prescription-only, or in CH where healthcare costs are high. In an OTC setting, more men experiencing ED visited a pharmacy; a majority of these men were recommended to visit a doctor for medical assessment. Given the high level of trust that patients place in a pharmacist’s advice, this opportunity should be leveraged for the early detection of possible underlying diseases (e.g., CV diseases) causing ED.

## Data Availability

Qualified researchers may request access to patient-level data and related study documents, including the study report and study protocol with any amendments, blank case report form, statistical analysis plan, and dataset specifications. Patient-level data will be anonymized, and study documents will be redacted to protect the privacy of participants. Further details on Opella’s data sharing criteria, eligible studies, and process for requesting access can be found at https://www.vivli.org/.
