# Supplementary material for: Exploring Patient Pathways and Care Situations in Men With Erectile Dysfunction in Different PDE-5 Inhibitor Regulatory Settings
Source: Int J Public Health. 2025 Dec 15;70:1608529. doi: 10.3389/ijph.2025.1608529 (PMC12745287; doi:10.3389/ijph.2025.1608529)
Supplement: Supplementary file 1 [file Supplementaryfile1.docx]

**Table S1**: Various questions to assess sexual performance

| **Questions** | **Score** |
| --- | --- |
| How do you rate your confidence that you could get and keep an erection? | Very low: Score 1 |
|  | Low: Score 2 |
|  | Moderate: Score 3 |
|  | High: Score 4 |
|  | Very high: Score 5 |
| When you had erections with sexual stimulation, how often were your erections hard enough for penetration (entering your partner)? | No sexual activity: Score 0 |
|  | Almost never or never: Score 1 |
|  | A few times (much less than half the time): Score 2 |
|  | Sometimes (about half the time): Score 3 |
|  | Most times (more than half the time): Score 4 |
|  | Almost always or always: Score 5 |
| During sexual intercourse, how often were you able to maintain your erection after you had penetrated (entered) your partner? | Did not attempt intercourse: Score 0 |
|  | Almost never or never: Score 1 |
|  | A few times (much less than half the time): Score 2 |
|  | Sometimes (about half the time): Score 3 |
|  | Most times (more than half the time): Score 4 |
|  | Almost always or always: Score 5 |
| During sexual intercourse, how difficult was it to maintain your erection to completion of intercourse? | Did not attempt intercourse: Score 0 |
|  | Extremely difficult: Score 1 |
|  | Very difficult: Score 2 |
|  | Difficult: Score 3 |
|  | Slightly difficult: Score 4 |
|  | Not difficult: Score 5 |
| When you attempted sexual intercourse, how often was it satisfactory for you? | Did not attempt intercourse: Score 0 |
|  | Almost never or never: Score 1 |
|  | A few times (much less than half the time): Score 2 |
|  | Sometimes (about half the time): Score 3 |
|  | Most times (more than half the time): Score 4 |
|  | Almost always or always: Score 5 |

**Table S2**: Severity of ED as per a scoring system

|  |  | SC_Score SUM |
| --- | --- | --- |
| 1 | Mild ED | 17–21 |
| 2 | Mild-to-moderate ED | 12–16 |
| 3 | Moderate ED | 8–11 |
| 4 | Severe ED and sexually active | 1–7 AND NONE of the following codes selected (SC.3=1, SC.4=1, SC.5=1, SC.6=1) |
| 5 | Severe ED and not sexually active | 1–7 AND (SC.3=1 OR SC.4=1 OR SC.5=1 OR SC.6=1) |
| 6 | No ED | >21 |
